# Dietary and temporal partitioning facilitates coexistence of sympatric carnivores in the Everest region

**DOI:** 10.1002/ece3.9531

**Published:** 2022-11-22

**Authors:** Hua Zhong, Fengjiao Li, Juan José Díaz‐Sacco, Kun Shi

**Affiliations:** ^1^ Wildlife Institute, School of Ecology and Nature Conservation Beijing Forestry University Beijing China; ^2^ Eco‐Bridge Continental Beijing China

**Keywords:** camera trapping, carnivore coexistence, high‐throughput sequencing, molecular diet analysis, temporal interaction, trophic niche

## Abstract

Carnivores, especially top predators, are important because they maintain the structure and function of ecosystems by top‐down control. Exploring the coexistence between carnivores belonging to different ecological guilds can provide the data needed for the development of effective conservation strategies of endangered species. We used scats and camera traps to molecularly analyze the dietary composition of four predators that inhabit the Everest region and assess their activity patterns. Dietary analysis revealed 22 food Molecular Operational Taxonomic Units (MOTUs) of 7 orders and 2 classes. Snow leopard (*Panthera uncia*) and wolf (*Canis lupus*) had high dietary overlap (Pianka's index = 0.95), as they both mainly preyed on ungulates (%PR = 61%, 50%), while lynx (*Lynx lynx*) and red fox (*Vulpes vulpes*) mainly consumed small mammals (%PR = 62%, 76%). We observed lower dietary overlaps (Pianka's index = 0.53–0.70) between predators with large body size difference (snow leopard versus lynx, snow leopard versus red fox, wolf versus lynx, wolf versus fox), and dietary difference was significant (*p* < .01), proving dietary partitioning. In activity pattern analysis, predators exhibited higher temporal overlaps with the more frequently consumed prey species, showing that predator activity can be regulated by prey availability. We observed no obvious temporal avoidance between snow leopard and wolf because they had high activity overlap (Δ = 0.87). Red fox had the lowest coefficients of activity overlap with snow leopard and wolf (Δ = 0.60, 0.59), suggesting that fox tends to avoid snow leopard and wolf temporally. In this study, we revealed how dietary and temporal partitioning facilitates the coexistence of carnivores in Everest. These results will help to increase the understanding of coexistence mechanism of carnivore communities, and provide the scientific foundation for the conservation of wildlife living in the Qinghai‐Tibet Plateau.

## Graphical abstract

In this study, experimental, statistical, and descriptive approaches are used to test the ecological niche theory. Through trophic and temporal niches, we aim to reveal the mechanisms of coexistence within a carnivore community and with their prey species in the alpine ecosystem of the Everest region.

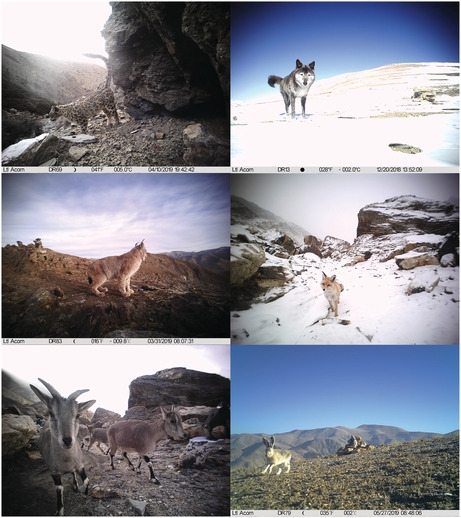

## 1 INTRODUCTION

Carnivore communities are important for maintaining the structure and function of terrestrial ecosystems. Living at the top of the food web, large carnivores maintain the populations of large herbivores at a stable level by exerting strong top‐down control on prey (Perilli et al., 2016; Schmitz et al., 2000), consequently stabilizing an ecosystem (Ripple & Beschta, 2012; Roemer et al., 2009). However, over the past two centuries, the global population of large carnivores has been seriously endangered because of habitat degradation, fragmentation, or shrinkage of prey populations (Ceballos & Ehrlich, 2002), making efforts for their research and conservation essential (Ripple et al., 2014). In the past 20 years, techniques such as camera trapping and high‐throughput sequencing have been extensively adopted to study interspecific interaction between carnivores, with a focus on dietary composition (Azevedo et al., 2006; Neale & Sacks, 2001; Ngoprasert et al., 2012) and/or habitat use of sympatric carnivores (Davis et al., 2021; Gehring & Swihart, 2003; Schuette et al., 2013).

Wolf and red fox are successful generalists, inhabiting various landscapes in the Northern Hemisphere (Díaz‐Ruiz et al., 2011; Krofel et al., 2022), while lynx occur in less disturbed areas of Eurasia. Comparatively, snow leopard are more threatened and only occur in Central Asia (Lyngdoh et al., 2020; Shrotriya et al., 2012). Interspecific competition among carnivores can be especially intense due to their large food requirements (Fedriani, Fuller, & York, 2000). Ecological theory predicts that sympatric carnivores should avoid competition through three ecological niches: trophic, temporal, and/or spatial (Gehring & Swihart, 2003; Lovari et al., 2015; Mccarthy et al., 2015).

In trophic niche, prey is the factor that could have the strongest influence on interspecific interaction among carnivores (Davis et al., 2021; Penido et al., 2017). Theoretically, there should be high dietary partitioning between small carnivores and the dominant carnivores to coexist in sympatry, yet small predator food habits have been reported to overlap considerably with those of larger predators (Paquet, 1992; Wang et al., 2014). As a mesocarnivore, a red fox may scavenge on the carrion left by a larger carnivore (Helldin & Danielsson, 2007), thus there can be considerable dietary overlap between small and large predators. Snow leopards are reported to specialize in hunting large prey, especially ungulates (Lu et al., 2019), while lynx generally consume medium‐sized or small prey (Sidorovich, 2006; Valdmann et al., 2005). Compared with felids, wolf and red fox are more generalized in diets (Castaeda et al., 2018; Lyngdoh et al., 2020). In temporal niche, mammal activity depends on various factors, such as abiotic factors (light intensity, temperature) related to astronomical events (sunrise, sunset, or zenith; Nouvellet et al., 2012), and biotic factors such as the presence of large apex predators and human activity (Azevedo et al., 2018; Vilella et al., 2020). In addition to food habits, competition among carnivores can be mitigated through temporal avoidance (Li et al., 2018).

The socio‐economic situation in the reserve underwent dramatic changes from 2000 to 2014: the human population increased by 28.9% and although the livestock population (mainly yak *Bos grunniens*, sheep *Ovis aries*, and goat *Capra hircus*) decreased by 9.9%, it remains one of the primary livelihood sources for local people and thus, grazing activity may pose potential threats to local wildlife (Chen, Gao, Wang, et al., 2016). Therefore, investigating how dependent predators are on livestock would provide sound scientific information, which will be helpful to reduce human‐carnivore conflict in the future.

In this study, we predicted that large‐sized predators (snow leopard and wolf) would coexist by temporal partitioning instead of consuming different prey. At the same time, predators with varying body sizes (red fox, lynx, vs. large‐sized predators) would coexist through dietary and temporal partitioning. Therefore, we aimed to (1) reveal the dietary compositions and trophic relationship of these carnivores by analyzing their diets with molecular techniques, (2) assess diel activity patterns and temporal relationship by analyzing camera‐trapping records, and (3) determine the importance of livestock in the diets of these predators. We hope these results will help to gain a comprehensive understanding into the mechanisms of the coexistence of sympatric carnivores in a heavily‐grazed alpine landscape.

## 2 METHODS

### 2.1 Study area

**FIGURE 1:**
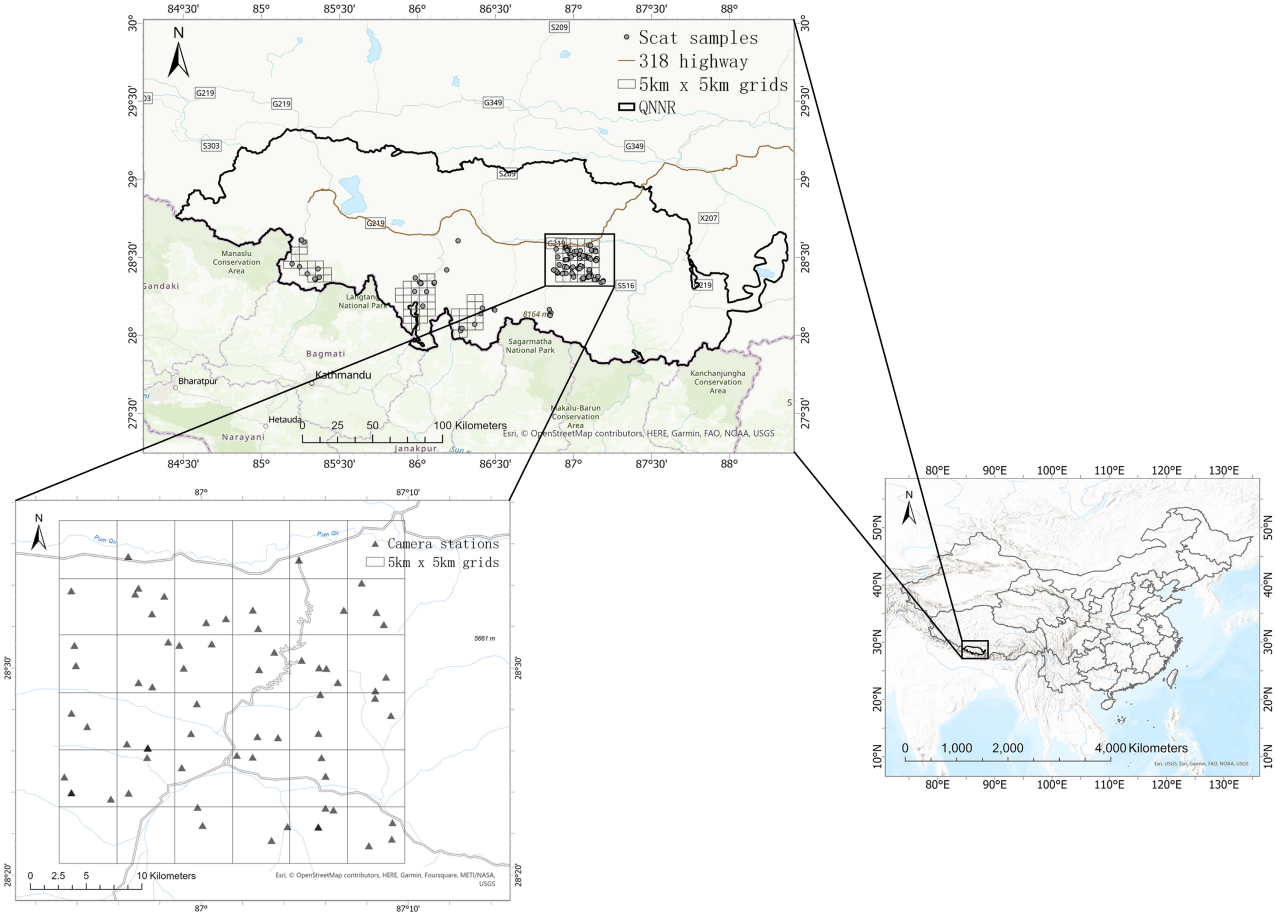
The location of QNNR (bottom right), the sampling area of scats (top), and the study area of camera trapping (bottom‐left).

The Qomolangma National Nature Reserve (from here, QNNR) of south Qinghai‐Tibet Plateau is located at the China–Nepal border (N: 27°48′—29°19′; E: 84°27′—88°21′), covering an area of more than 33,700 km^2^ (Figure 1). Holding the world's highest peaks, including Mount Everest (8848.86 m; Yamin et al., 2020) and 4 other peaks above 8000 m, QNNR stands as the reserve at the higher elevation in the world with an average altitude of above 4000 m (Hu et al., 2014). Although carnivores have been studied extensively on the southern slopes of Mount Everest in Nepal (Ale et al., 2007; Chetri et al., 2017; Lovari et al., 2013; Oli et al., 1994; Schuette et al., 2013), little is known about the carnivore community in areas to the North of the High Himalayas, especially how these species can coexist in such a harsh plateau environment. Despite the fact that high altitudes present some of the harshest environments in the world, QNNR harbors one of the world's richest carnivore assemblages (Ripple et al., 2014), including snow leopard *Panthera uncia*, gray wolf *Canis lupus*, Eurasian lynx *Lynx lynx*, red fox *Vulpes vulpes*, Pallas's cat *Otocolobus manul*, and leopard *Panthera pardus*, etc. (Chen, Gao, Wang, et al., 2016; Hu et al., 2014; Jackson et al., 1995). Other mammals, such as bharal (*Pseudois nayaur*), plateau hare (*Lepus oiostolus*), and Himalayan marmot (*Marmota himalayana*), are very commonly see in the reserve (Hu et al., 2014).

**FIGURE 2:**
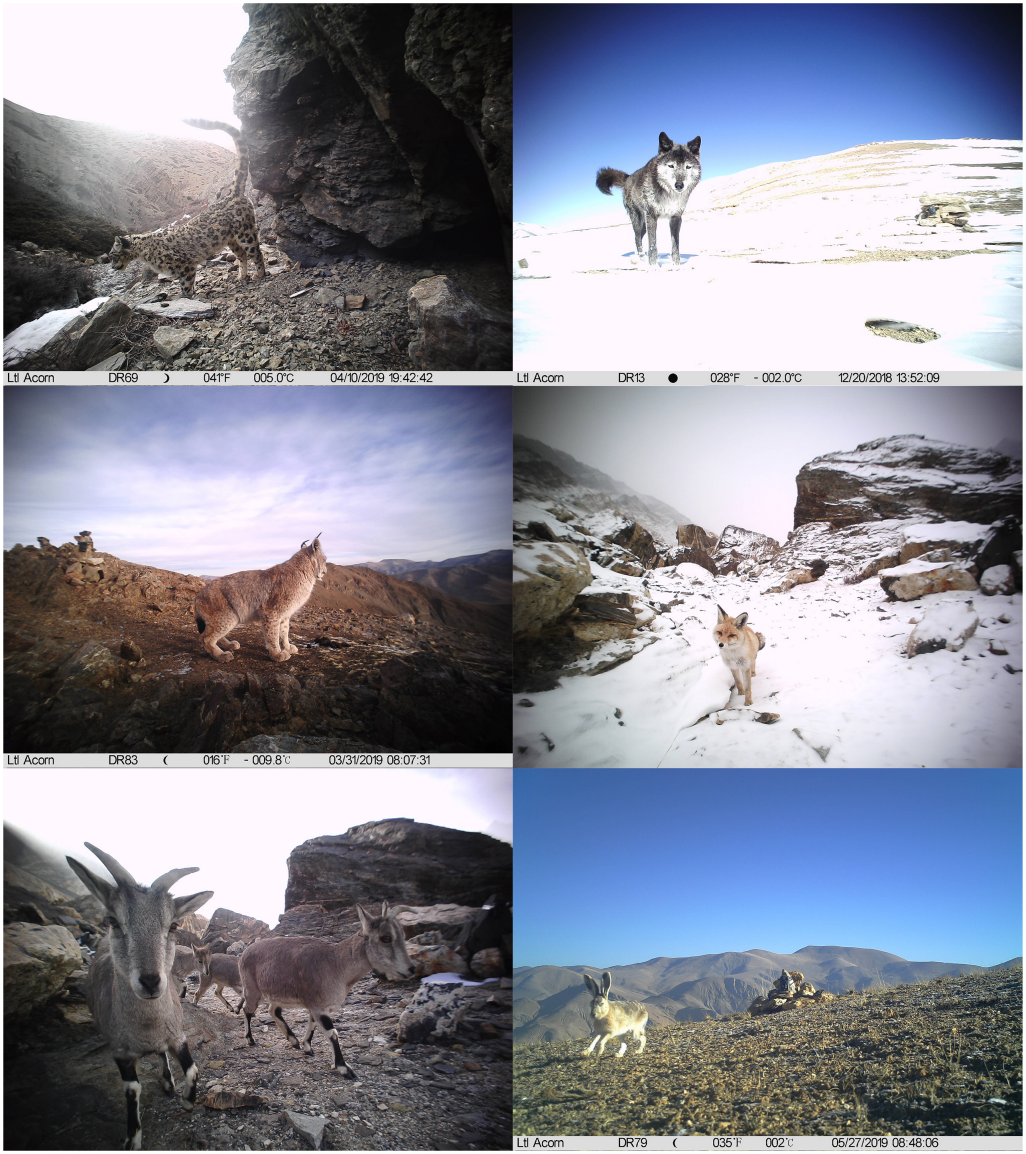
Example of camera‐trapping pictures obtained during the study. From top left to bottom right: Snow leopard *Panthera uncia*, wolf *Canis lupus*, lynx *Lynx lynx*, red fox *Vulpes vulpes*, bharal *Pseudois nayaur*, and plateau hare *Lepus oiostolus*.

Our study was conducted in the northern aspect of QNNR, which harbors populations of carnivores such as snow leopard, wolf, lynx, and red fox (Figure 2). The main ecosystem is alpine meadow or bare rock, with an average altitude of about 4500 m. In November 2017, a camera‐trapping survey was conducted in a remote area of Zhaxizong and Zhaguo of Dingri county, where 118 infrared cameras (113 Ltl Acorn and 5 RECONYX™) were systematically installed in an area of 900 km^2^ divided into 36 grids of 5 × 5 km. Transects of at least 5 km were covered in each grid to deploy cameras and collect scats, depending on accessibility. For each camera station, two cameras were set, face‐to‐face at a slight angle. In the next 3 years, cameras were revisited and maintained for battery and SD card replacement about every half year. All the cameras were finally retrieved in July 2020. Scat samples from 2017 to 2019 were collected in the same camera‐trapping area mentioned above, while scats from July 2020 were collected in three valleys of Nielamu, Jilong, and Dingri county (Figure 1).

### 2.2 Dietary analysis

Each scat was sampled by removing its tip and placing it in a 50 ml centrifuge tube with 30 ml silica beads in order to keep it dry (Wasser et al., 1997). Scat samples remained under the condition of shading, low temperature, and stored at minus 20°C after the field survey. When DNA was extracted for the first time, primers ATP6 (Chaves et al., 2011), and 16S (Xiong et al., 2016; Appendix S1: Table S2) were used to identify the carnivore species. After a homogenization process, DNA was extracted for the second time to identify the prey species, and amplified with tagged universal primers 12SV5 (Riaz et al., 2011; Appendix S1: Table S2). Blocking oligos (Hege & Simon, 2008; Vestheim et al., 2011; Shao et al., 2019; Appendix S1: Table S3) were used to block the amplification of predator DNA. PCR products were purified, pooled, and sequenced on NovaSeq6000 (Illumina Inc., San Diego, CA, USA). The resulting sequencing reads were filtered, processed by QIIME 2 (2022.02) (Bolyen et al., 2019), and sorted into the corresponding samples. Prey species were determined by using BLAST (Altschul, 2012), based on the local species inventory, “A Guide to the Mammals of China” (Smith et al., 2010), “A Field Guide to the Birds of China” (Mackinnon & Phillipps, 2000), and the IUCN Red List. Detailed information about the methods is in Appendix S1.

For each sample, a MOTU (Molecular Operational Taxonomic Unit) is assigned a value of 1 when it is present, and 0 when it is not (Pompanon et al., 2012). Dietary composition based on MOTU was statistically analyzed as follows (Lovari et al., 2010): (1) Occurrence frequency (%FO) and relative proportion (%PR) were adopted to quantify the dietary composition of each carnivore species. For a specific predator, %FO is the percentage of (the occurrence frequency of a particular MOTU)/(the number of effective samples), indicating how common the prey is; %PR is the percentage of (the occurrence frequency of a particular MOTU)/(the occurrence frequency of all MOTUs), indicating the degree of importance of this prey species (Pompanon et al., 2012). (2) We quantified the degree of interspecific dietary overlap at the level of class taxa using Pianka overlap index (Pianka & Pianka, 1976), which varies from 0 (no overlap) to 1 (complete overlap). (3) Interspecific dietary differences at the class level were statistically analyzed using the Fisher's exact test with a level of 0.05.

Predators were sorted in order of body mass based on previous literature (Chundawat, 1992; Shrotriya et al., 2012; Smith, Lyons, et al., 2003; Smith, Peterson, & Houston, 2003). According to the size of the prey species, we divided all the MOTUs as follows: Artiodactyla and Perissodactyla are classified as “Ungulate mammals”; Lagomorpha and Rodentia are classified as “Small mammals”; bird species are classified as “Large birds” (Galliformes) and “Small birds” (Passeriformes, Strigiformes; Figure 4).

### 2.3 Activity pattern analysis

We adopted the R package “overlap” presented by Ridout & Linkie (2009) to fit the kernel density curve and determine the temporal overlap. Prior to analysis, we adjusted clocktime of each independent event to suntime (sunrise or sunset) using “suntime” function in “overlap” package (Nouvellet et al., 2012). Percentage of diurnal/nocturnal activity was derived from the proportion of independent events recorded during the day (from sunrise to sunset) or at night (from sunset to sunrise; van Schaik & Griffiths, 1996). Overlapping coefficient of Δ_4_ (hence Δ) (Ridout & Linkie, 2009) was used to quantify the degree of temporal overlap, ranging from 0 (no overlap) to 1 (identical activity patterns), and a smoothed bootstrap of 1000 iterations was operated and the basic0 output was chosen as 95% confidence intervals (Ridout & Linkie, 2009). According to preliminary work (Appendix S2: Figure S1), the activity patterns of human (*n* = 1305), dog (*n* = 873), sheep and/or goat (*n* = 1939), and yak (*n* = 259) were quite similar, and they were pooled together to represent anthropogenic factors. Detailed information about the methods is in Appendix S1.

## 3 RESULTS

### 3.1 Dietary composition

A total of 234 scat samples were collected in QNNR from Oct. 2017 to Dec. 2020, and 220 of them were identified at the species level (94%). Most of the scats were from snow leopard (*n* = 42), wolf (*n* = 54), dog (*n* = 44), lynx (*n* = 30), and red fox (*n* = 42). A few were from Pallas's cat (*n* = 4), and leopard cat (*n* = 2), which were excluded from the analysis. A total of 0.33G clean data were filtered from 6.84G raw data from high‐throughput sequencing across 5 libraries for further analysis. A total of 22 MOTUs were identified, including 15 mammalian MOTUs and 7 bird MOTUs (Appendix S1: Table S1), belonging to 2 classes and 7 orders.

**FIGURE 3:**
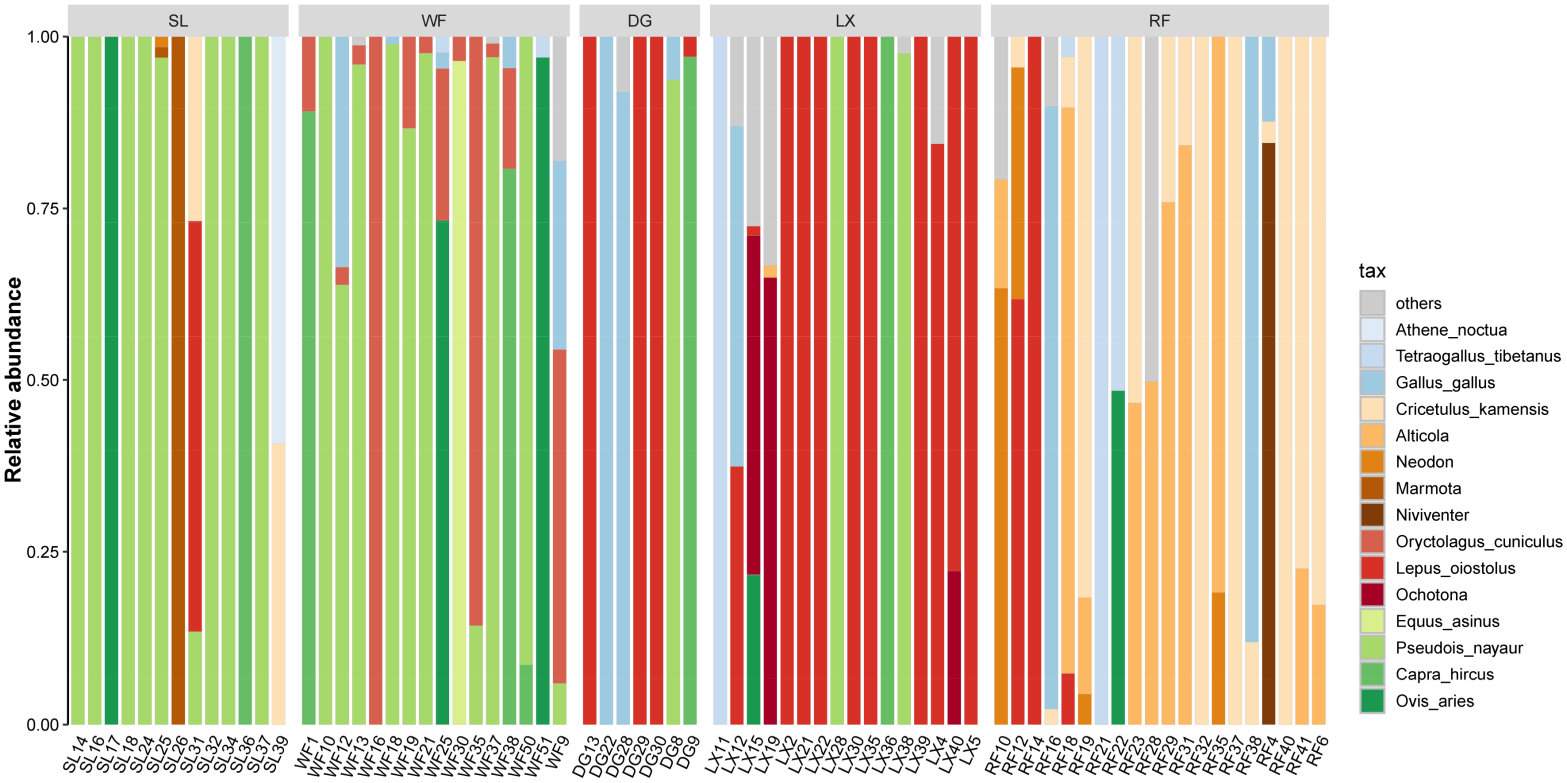
The relative abundance of different food MOTUs (based on OUT abundance) in the diet of five predators (DG, dog; LX, lynx; RF, red fox; SL, snow leopard; WF, wolf). Seven MOTUs (Molecular Operational Taxonomic Units) of least reads were pooled in “others.”

Eight food MOTUs were identified in the snow leopard's diet, with an average of 1.4 MOTUs per sample. Bharal (Figure 3) was found to be the most frequent prey species (%FO = 69%). Eight food MOTUs were found in the wolf's diet, 2.4 MOTUs per sample, and bharal were the most frequently consumed ungulate species (%FO = 63%). There were 12 food MOTUs in the lynx's diet, 1.6 MOTUs per sample. Plateau hare was the most frequently consumed prey species (%FO = 75%). We found the most prey species, namely 13 food MOTUs in the diet of red fox, with an average of 2.1 MOTUs per sample. Dog consumed 5 MOTUs in total, with an average of 1.4 MOTUs per sample. Plateau hare and chicken (*Gallus gallus*) were found to be the most frequently consumed prey species. Dog was excluded from subsequent dietary analysis due to its small effective sample size.

**FIGURE 4:**
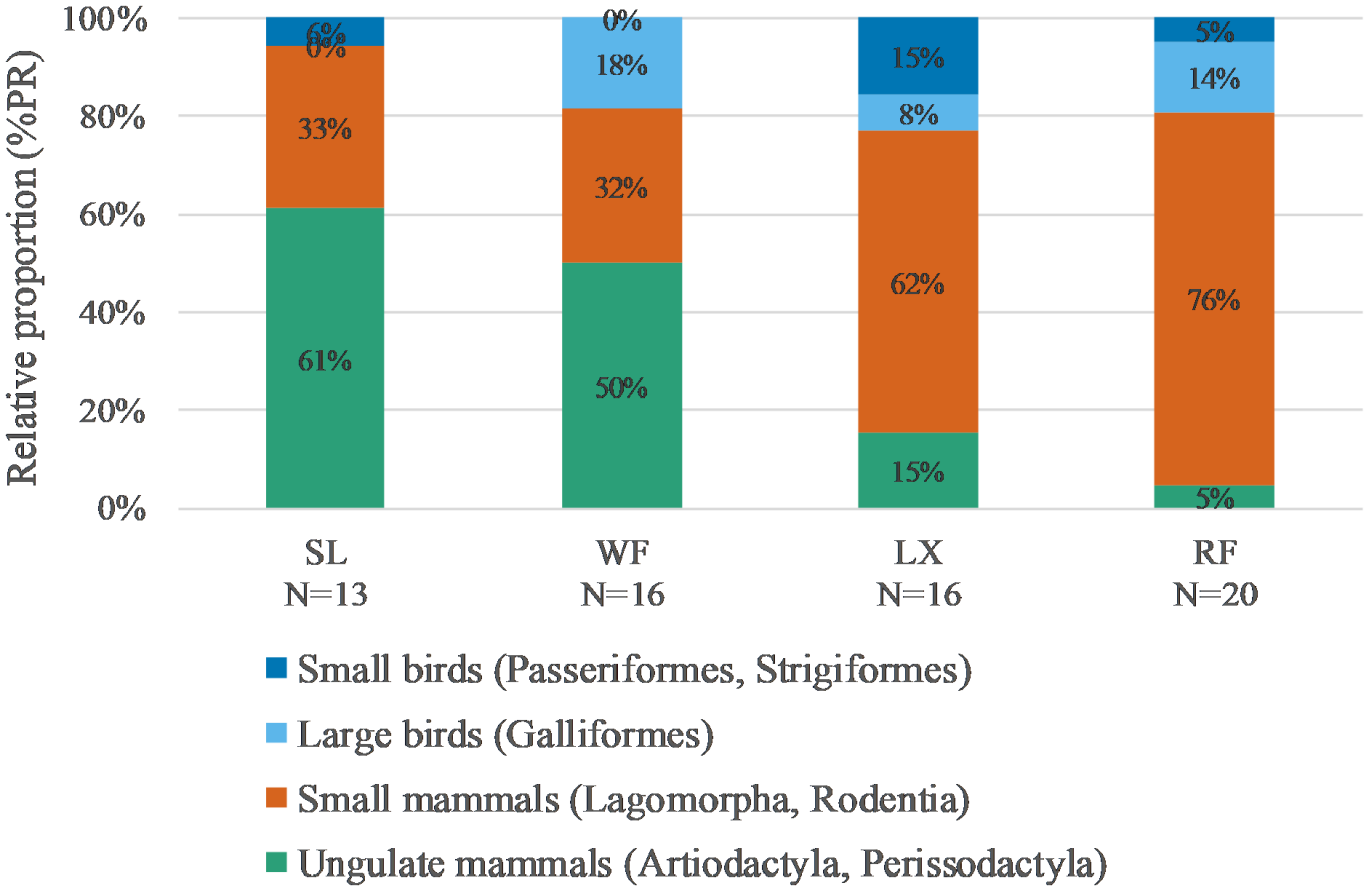
The relative proportion (%PR) of mammals and birds in the diet of four wild predators (LX, lynx; RF, red fox; SL, snow leopard; WF, wolf).

Snow leopard and wolf both consumed more ungulates (%PR = 61%, 50%) and less small mammals (%PR = 33%, 32%), while lynx and red fox primarily preyed on small mammals (%PR = 62%, 76%; Figure 4). Regarding livestock, snow leopard (%FO = 15%, %PR = 11%), lynx (%FO = 13%, %PR = 8%), and red fox (%FO = 10%, %PR = 5%) consumed less livestock less than wolf (%FO = 38%, %PR = 16%).

### 3.2 Dietary comparison

Higher dietary overlaps were observed between snow leopard and wolf (Pianka's index = 0.95), lynx and fox (Pianka's index = 0.97; Table 1). Lower dietary overlaps were observed between predators with large body size differences (snow leopard versus lynx, snow leopard versus red fox, wolf versus lynx, wolf versus fox; Pianka's index = 0.53–0.70).

**TABLE 1:** Pianka's dietary overlap (Δ) index values between carnivore species (LX, lynx; RF, red fox; SL, snow leopard; WF, wolf) in the Everest region.

Predators	SL	WF	LX	RF
SL	/	0.95	0.67	0.53
WF	/	/	0.70	0.60
LX	/	/	/	0.97
Body mass(kg)	36–52^a^	35^b^	17.75^c^	6.33^c^

*Note*: The body mass data were based on previous literature: ^a^(Chundawat, 1992), ^b^(Shrotriya et al., 2012), ^c^(Smith, Peterson, & Houston, 2003).

The Fisher's exact test shows (Table 2) that diets of predators with large body size differences (snow leopard versus lynx, snow leopard versus red fox, wolf versus lynx, wolf versus fox) were at least significantly different (*p* < .01), while no statistical differences (*p* > .05) of diets were found between snow leopard and wolf, lynx and fox.

**TABLE 2:** Fisher's exact test statistics of dietary composition between carnivore species (LX, lynx; RF, red fox; SL, snow leopard; WF, wolf) in the Everest region.

Predators	SL	WF	LX	RF
SL	/	H_0_	**	***
WF	/	/	***	***
LX	/	/	/	H_0_

H_0_: *p* > .05, *p < .05, **p < .01, ***p < .001.

### 3.3 Activity pattern comparison

A total of 118 cameras were systematically deployed at 67 camera stations in 31 grids in November. 2017. After 3 years of camera trapping, 12,168 independent events were obtained, including snow leopard (*n* = 379), wolf (*n* = 536), lynx (*n* = 307), red fox (*n* = 793), bharal (*n* = 1430), hare (*n* = 4347), and anthropogenic factors (*n* = 4376; Table 3).

**TABLE 3:** Estimates of coefficients of overlapping (Δ), 95% confidence intervals (CI), and number of independent events (*n*), originating from camera trapping (LX, lynx; RF, red fox; SL, snow leopard; WF, wolf), in the Everest region. Human, dog, sheep, and yak events were pooled together in “Anthropogenic factors” based on preliminary analysis (Appendix S1: Figure S1) since their daily activity patterns were similar.

Species	*n*	SL		WF		LX		RF	
		*n* = 379		*n* = 536		*n* = 307		*n* = 793	
		Δ	CI	Δ	CI	Δ	CI	Δ	CI
Prey									
Bharal	1430	0.62	0.57–0.67	0.68	0.63–0.72	0.47	0.42–0.51	0.26	0.23–0.29
Hare	4347	0.75	0.71–0.79	0.70	0.67–0.74	0.90	0.87–0.94	0.79	0.77–0.82
Anthropogenic factors									
Human, sheep, yak, dog	4376	0.51	0.47–0.56	0.57	0.53–0.61	0.37	0.30–0.39	0.18	0.17–0.21

**FIGURE 5:**
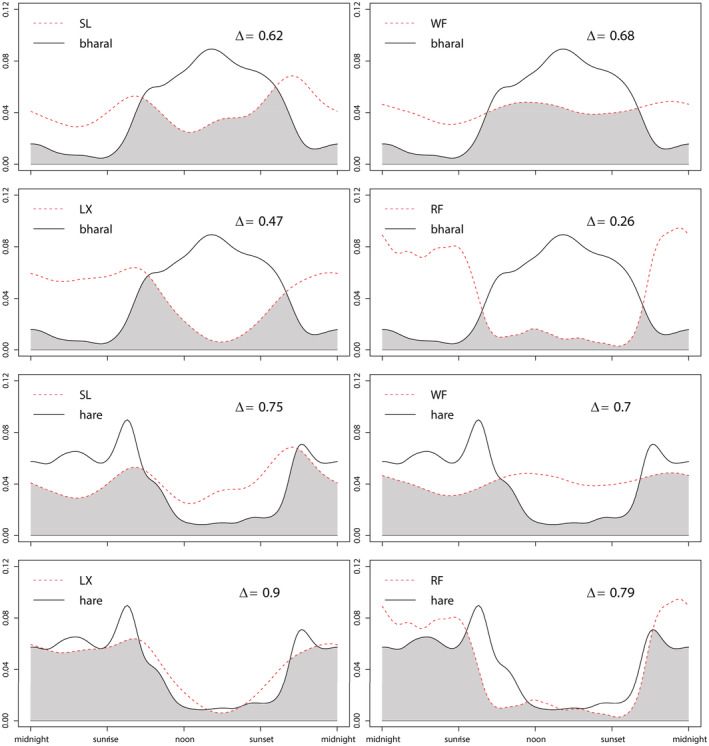
Daily activity patterns of bharal/hare and wild predators (LX, lynx; RF, red fox; SL, snow leopard; WF, wolf), indicated by kernel density curves, and pairwise overlaps between them. Values on the y‐axis represent ‘density of activity’.

Bharal exhibited 75% diurnal activity and 25% nocturnal activity. Four predators overlapped differently with bharal, as red fox had lower overlaps (Δ = 0.26), and snow leopard and wolf had higher overlaps (Δ = 0.62, Δ = 0.68; Figure 5).

Lynx and hare had rather similar activity patterns: they both exhibited less diurnal activity (36%, 37%) and more nocturnal activity (64%, 63%). And lynx had the highest overlapping coefficient with hare (Δ = 0.90; Figure 5).

**FIGURE 6:**
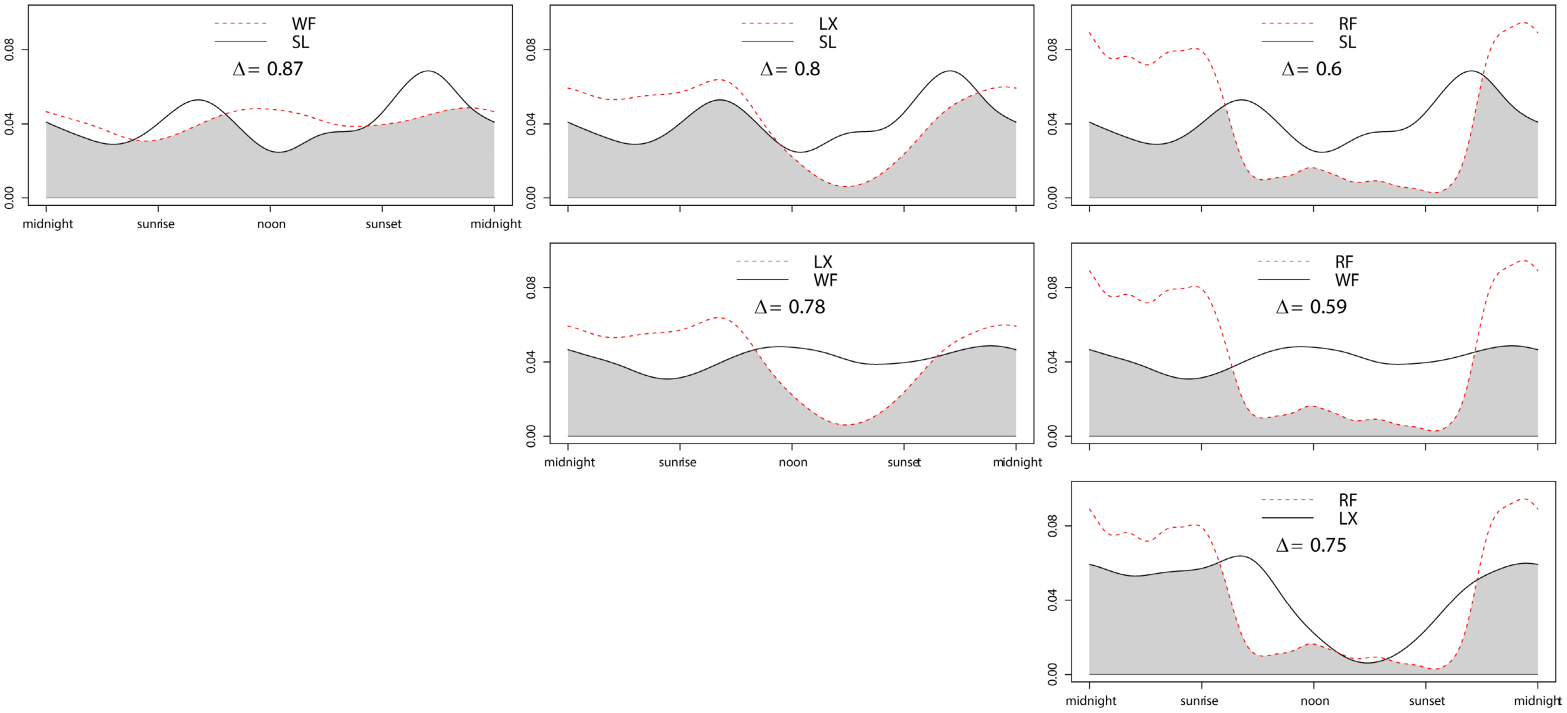
Daily activity patterns of predators (LX, lynx; RF, red fox; SL, snow leopard; WF, wolf), and pairwise overlaps between them. Values on the y‐axis represent ‘density of activity’.

Snow leopard and wolf had rather similar activity patterns. Snow leopard exhibited 46% diurnal activity and 54% nocturnal activity and had two clear activity peaks at sunrise and sunset, suggesting that it was predominantly crepuscular. Wolf exhibited 52% diurnal activity and 48% nocturnal activity and had two mild peaks near noon and midnight, respectively. Therefore, snow leopard and wolf had a higher overlap (Δ = 0.87). The activity patterns of red fox, being mostly nocturnal (77%), were not similar to those of snow leopard and wolf and red fox had lower overlaps with snow leopard and wolf (Δ = 0.60, 0.59; Figure 6).

**FIGURE 7:**
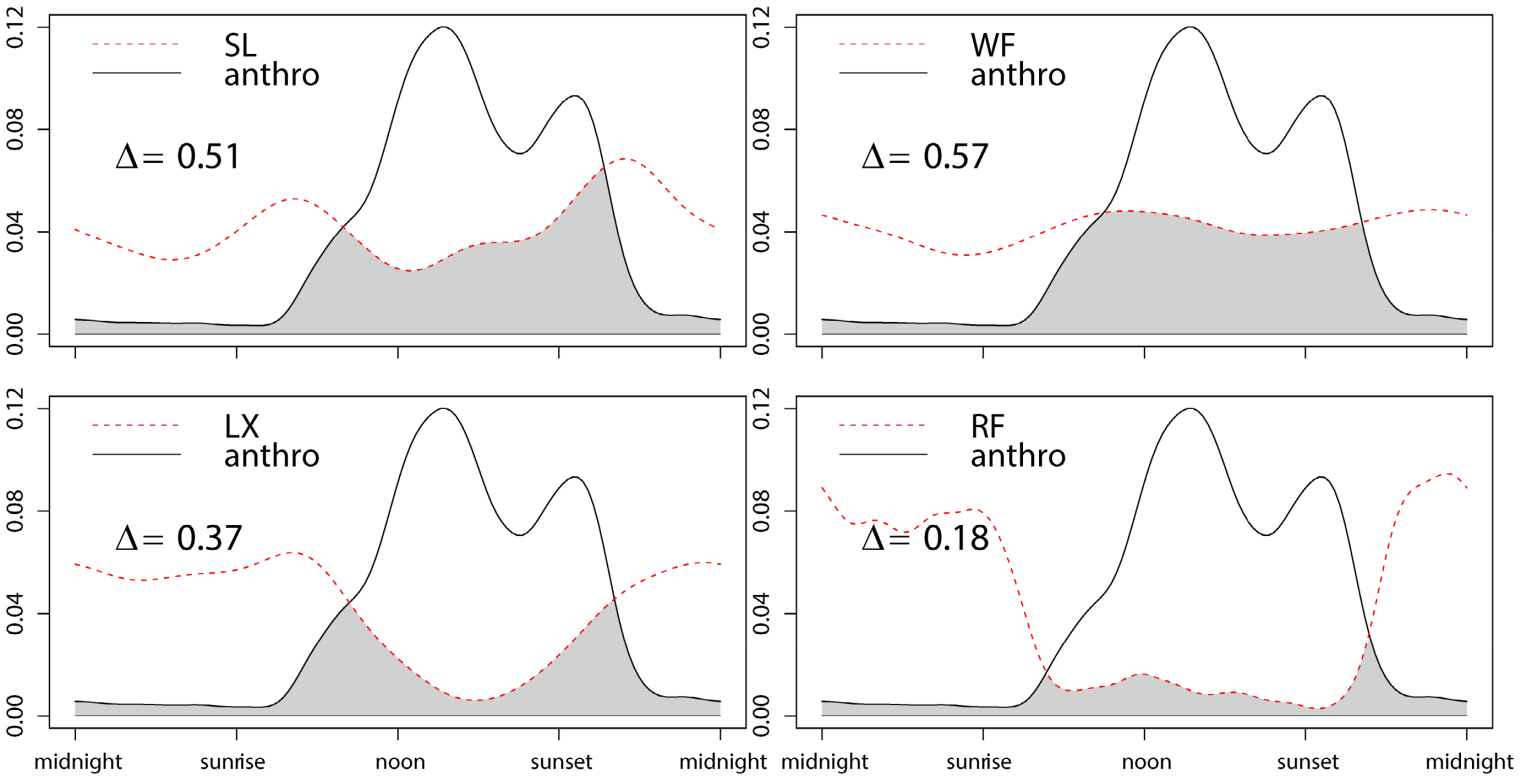
Daily activity patterns of anthropogenic factors (human, dog, sheep, yak) and wild predators (LX, lynx; RF, red fox; SL, snow leopard; WF, wolf), and pairwise overlaps between them. Values on the y‐axis represent ‘density of activity’.

Activities related to anthropogenic factors were detected predominantly during the day (75%) with a near‐noon activity peak and a sunset peak. Snow leopard, wolf, and lynx had higher temporal overlaps with anthropogenic factors (Δ = 0.51, 0.57, 0.37), while red fox presented lower overlaps (Δ = 0.18; Figure 7).

## 4 DISCUSSION

### 4.1 Niche overlaps between top predators

There was no obvious dietary partitioning between snow leopards and wolves. High interspecific dietary overlap was observed between them (Table 1), and their diets had no statistical differences (Table 2). Similar‐sized predators can prey on similar prey species, thus their diets could be similar (Lovari et al., 2013), implying that they do not base their coexistence on diet partitioning. In temporal analysis, snow leopards and wolves had similar activity patterns, and they were both found to overlap more with bharal, compared with lynx and red fox. This is in line with our dietary analysis that identified snow leopard and wolf as preying more on bharal than smaller predators. We believe this temporal behavior adaptation enables snow leopards and wolves more opportunities to capture bharals.

Additionally, we found that the activity pattern of bharal seems more diurnal, which is partly inconsistent with previous studies, in which bharals identified as crepuscular animals, were reported to have two feeding peaks at dawn and dusk (Schaller, 1972), and spend most noon hours resting and bedding (Long et al., 2009). This inconsistency can be explained by the difference between detection‐time data and motion intensity data yielded, respectively, from camera trapping and radio/GPS collar: camera traps are programmed to record when animals are present even when they are inactive (for example, resting; Ridout & Linkie, 2009).

Contrary to our prediction, we found that there was no clear dietary or temporal partitioning between snow leopards and wolves since they presented both high dietary overlap and high temporal overlap. This might suggest that spatial partitioning could play a major role in the coexistence of these two sympatric predators (Lovari et al., 2013), or that the food resources (mainly ungulates) are abundant. We acknowledge that further investigation of interspecific relationships in the spatial dimension will give us a more thorough understanding of the coexistence mechanisms of these two large predators. As opportunistic, ambush predators, snow leopards are reported to typically prefer steep and rugged terrain (Jackson & Ahlborn, 1989), while wolves are cursorial predators that are highly adapted to chasing their prey over long distances (Husseman et al., 2003). It could be hypothesized that the difference in hunting strategies might lead to different habitat use, facilitating spatial separation and coexistence between the two apex predators.

### 4.2 Niche differentiation promotes coexistence

Our study is the first to focus on carnivore coexistence in the northern aspect of the High Himalayas. In the dietary analysis, we found that lynx and red fox in the Everest had lower dietary overlaps with snow leopard and wolf. This is because lynx and red fox mainly consumed lagomorphs and rodents, respectively, while snow leopard and wolf mainly preyed on ungulates (Figure 4). Therefore, our study supports the conclusion that large carnivores could be more specialized in preying on large ungulates due to their high energy profitability (Lanszki et al., 2019), while mesocarnivores could have more generalized food habits (Shao et al., 2021), mainly preying upon small mammals. Additionally, we found that lynx had the highest temporal overlapping coefficient with hare. This is also in line with our dietary analysis and previous studies that identified the hare as one of the most important preys of lynx. (Burstahler et al., 2016; Sidorovich, 2006; Valdmann et al., 2005). According to our classification criteria, lynx and red fox had a high dietary overlap, since lagomorphs and rodents are both classified as “small mammals.” However, at the Order level, they had a much lower dietary overlap (Pianka's index = 0.21), and a significant dietary difference was observed (*p* < .01).

Our results also showed trends that (1) diet overlap was negatively related to body size difference (Table 1), (2) diet difference was positively related to body size difference (Table 2). Meanwhile, the diets of predators with large body size differences were significantly different, consequently, we proved the existence of dietary partitioning and concluded that dietary partitioning facilitates the coexistence between large and smaller carnivores. However, dominant predators may also eliminate competition with smaller predators by killing them (Fedriani, Fuller, Sauvajot, & York, 2000; Hass, 2009; Palomares & Caro, 1999). Therefore, the latter is likely to avoid the former spatially and/or temporally (Bu et al., 2016; Ramesh et al., 2012). In the diel rhythms analysis, activity patterns of red foxes were found to be nocturnal, which were different from those observed in both snow leopards and wolves (Figure 6). At the same time, red foxes had lower temporal overlaps with snow leopards and wolves (Table 3), suggesting that red foxes tend to avoid the dominant predators temporally. Overall, we conclude that, dietary partitioning, together with temporal partitioning, could facilitate coexistence between large predators and smaller predators.

### 4.3 Human‐wildlife conflict

Since the 1990s, poaching and illegal trade in the Mount Everest region have decreased substantially (Jackson et al., 1995), which may be largely attributed to the implementation of conservation efforts such as a grazing restriction policy, confiscation of firearms, strong law enforcement, environmental public education, and ecological compensation programmes (Chen, Gao, Wang, et al., 2016). These effective conservation measures have allowed carnivore populations to recover. Livestock depredation by large carnivores was frequently reported in the QNNR. Interestingly, Jackson et al. (1995) tend to blame the depletion of wild prey on lax animal husbandry practices, but local residents in QNNR generally attributed the problem to carnivore population growth, notably wolf recovery (Chen, Gao, Lee, et al., 2016). Fortunately, they also found that local people generally support carnivore conservation, with only a small minority holding negative attitudes (Chen, Gao, Lee, et al., 2016; Chen, Gao, Wang, et al., 2016).

A previous study determined that wolf, lynx, and snow leopard were found to be the major livestock predators in QNNR (Chen, Gao, Lee, et al., 2016). This discovery agrees with our study which found that wolf, lynx, and snow leopard had higher temporal overlaps with herding activity, compared with red fox. According to our dietary analysis, carnivores generally consumed domestic animals (goat, sheep, yak, mule *Equus asinus*) at low levels. We also found that livestock was most frequently detected and most important in the diet of wolf. This result also coincides with the discovery that found wolf to be the species that causes the most conflicts with local residents (Chen, Gao, Lee, et al., 2016). From 2011 to 2013, total livestock loss accounted for 1.2% of the entire stockholding (*n* = 846,707) in the region (Chen, Gao, Lee, et al., 2016). To prevent the potential escalation of human‐wildlife conflicts and resurgence of retaliatory killing of large predators, we suggest preventing lax animal husbandry practices and severe depredation (when 10 to 50 or more sheep and/or goat were killed in a single incident) through measures such as fence reinforcement. At the same time, conservation implications that teach locals about the ecological significance of carnivore communities are also needed to foster continuous widespread positive attitudes towards them.

## 5 CONCLUSION

In this study, we revealed the dietary composition and activity pattern of a carnivore community in the Everest region. We also discovered the trophic relationships and temporal interaction between these carnivores and their main preys, demonstrating that large carnivores in the Everest rely heavily on ungulates. Therefore, we would like to highlight the importance of improving conservation schemes for these herbivore species that are under grazing pressure. Finally, we revealed how dietary and temporal partitioning facilitate the coexistence of carnivores. These results will help to increase understanding of carnivore communities, and provide the scientific foundation for the conservation of threatened species in the Mount Everest region.

## AUTHOR CONTRIBUTIONS

**Hua Zhong:** Conceptualization (lead); data curation (lead); formal analysis (lead); funding acquisition (supporting); investigation (lead); methodology (lead); project administration (supporting); resources (supporting); software (lead); supervision (lead); validation (lead); visualization (lead); writing – original draft (lead); writing – review and editing (lead). **Fengjiao Li:** Conceptualization (supporting); data curation (equal); formal analysis (equal); funding acquisition (supporting); investigation (supporting); methodology (supporting); project administration (supporting); resources (supporting); software (equal); supervision (supporting); validation (supporting); visualization (equal); writing – original draft (supporting); writing – review and editing (supporting). **Juan José Díaz‐Sacco:** Conceptualization (equal); data curation (equal); formal analysis (supporting); funding acquisition (supporting); investigation (supporting); methodology (equal); project administration (supporting); resources (supporting); software (supporting); supervision (supporting); validation (equal); visualization (supporting); writing – original draft (supporting); writing – review and editing (equal). **Kun Shi:** Conceptualization (equal); data curation (equal); formal analysis (equal); funding acquisition (lead); investigation (equal); methodology (equal); project administration (lead); resources (lead); software (supporting); supervision (lead); validation (supporting); visualization (supporting); writing – original draft (supporting); writing – review and editing (supporting).

## CONFLICT OF INTEREST

The authors declare no conflict of interest and note that a part of the scat samples in this study (2017 out of all) was used by Zhixiong Deng in his undergraduate thesis (unpublished), and the corresponding data of scat species identification was included in this study. Similarly, a part of camera‐trap data (2017 out of all) was used by Changxi Xiao in his published journal article. The rest of the data is original to the study and has not been used in other works that are published, in press, submitted, or soon to be submitted elsewhere.

## Supporting information


Appendix S1.

## Data Availability

Data in this study (raw data used to generate tables, figures, plots) have been permanently archived in Dryad repository (Dietary and temporal partitioning facilitates coexistence of sympatric carnivores in the Everest region). All methods or protocols utilized to generate the data have been cited in the article or supporting information. This study includes no novel code, computer software or derived data products.
