# Supplementary material for: Dietary and temporal partitioning facilitates coexistence of sympatric carnivores in the Everest region
Source: Ecol Evol. 2022 Nov 22;12(11):e9531. doi: 10.1002/ece3.9531 (PMC9682211; doi:10.1002/ece3.9531)
Supplement: Supplementary file 1 — Appendix S1. [file ECE3-12-e9531-s001.docx]

**Hua Zhong *et al*.**

Dietary and temporal partitioning facilitate sympatric coexistence of carnivore assemblage in Everest region.

**Appendix S1-** **MOTU Table**

Appendix S1: Table S1 Result of prey MOTUs of five carnivores identified by molecular dietary analysis.

| Prey MOTU | Occurrence in different carnivore samples | | | | | Best match species in GenBank | | |
| --- | --- | --- | --- | --- | --- | --- | --- | --- |
| Scientific name | SL  *N*=13 | WF  *N*=16 | DG  *N*=7 | RF  *N*=20 | LX  *N*=16 | Scientific name | Best  identity(%) | Accession |
| Artiodactyla | | | | | | | | |
| *Pseudois nayaur*  *Sus scrofa*  *Capra hircus*  *Ovis aries*  *Bos grunniens*  *Ovis ammon* | 9 | 10 | 1 | 0 | 1 | *Pseudois nayaur* | 100% | KP998469 |
|  | 0 | 3 | 0 | 0 | 0 | *Sus scrofa* | 100% | OM069301 |
|  | 1 | 3 | 1 | 0 | 1 | *Capra hircus* | 100% | MZ073671 |
|  | 1 | 2 | 0 | 1 | 1 | *Ovis aries* | 100% | KU681175 |
|  | 0 | 0 | 1 | 1 | 0 | *Bos grunniens*  *Bos taurus* | 100%  100% | OK271109  MZ901759 |
|  | 0 | 0 | 0 | 0 | 1 | *Ovis ammon* | 100% | KT781689 |
| Perissodactyla | | | | | | | | |
| *Equus asinus* | 0 | 1 | 0 | 0 | 0 | *Equus asinus* | 100% | NC_001788 |
| Lagomorpha | | | | | | | | |
| *Lepus oiostolus* | 1 | 0 | 4 | 3 | 12 | *Lepus oiostolus* | 100% | NC_050983 |
| *Oryctolagus cuniculus* | 0 | 12 | 0 | 0 | 0 | *Oryctolagus cuniculus* | 99.2% | NC_001913 |
| *Ochotona* | 0 | 0 | 0 | 0 | 3 | *Ochotona cansus*  *Ochotona thibetana*  *Ochotona curzoniae* | 99.1%  99.1%  99.1% | MN547482  MN547429  MN547423 |
| Rodentia | | | | | | | | |
| *Cricetulus kamensis* | 2 | 0 | 0 | 14 | 0 | Cricetulus kamensis | 100% | MH159227 |
| *Alticola* | 0 | 0 | 0 | 10 | 1 | *Myodes rutilus*  *Myodes rufocanus* | 99.2%  99.2% | MK482363  KT725595 |
| *Marmota* | 2 | 0 | 0 | 0 | 0 | *Marmota monax*  *Marmota marmota* | 96.4%  95.5% | LR632920  MN935776 |
| *Neodon* | 1 | 0 | 0 | 4 | 0 | *Microtus richardsoni* | 100% | NC_049220 |
| *Niviventer* | 0 | 0 | 0 | 1 | 0 | *Niviventer confucianus*  *Niviventer culturatus*  *Niviventer sacer* | 96.5%  95.6%  95.6% | MH283869  OM334919  MZ935252 |
| Galliformes | | | | | | | | |
| *Tetraogallus tibetanus* | 0 | 2 | 0 | 3 | 1 | *Tetraogallus tibetanus* | 100% | KF027439 |
| *Gallus gallus* | 0 | 5 | 3 | 3 | 1 | *Gallus gallus* | 100% | OL689234 |
| Passeriformes | | | | | | | | |
| *Alaudidae* | 0 | 0 | 0 | 1 | 0 | *Eremophila alpestris*  *Calandrella cinerea* | 100%  100% | NC_048470  NC_048469 |
| *Passeriformes* | 0 | 0 | 0 | 1 | 1 | *Sturnella magna*  *Loxia leucoptera*  *Agelaius phoeniceus*  *Neospiza concolor* | 100%  100%  100%  100% | U86140  NC_051015  MN356439  KY078925 |
| *Passeroidea* | 0 | 0 | 0 | 0 | 2 | *Passer domesticus*  *Passer montanus*  *Petronia petronia*  *Leucosticte brandti* | 100%  100%  100%  100% | MW524523  MH211399  MF071218  KM078775 |
| *Aegithalos* | 0 | 0 | 0 | 0 | 1 | *Aegithalos caudatus*  *Aegithalos concinnus*  *Aegithalos glaucogularis*  *Aegithalos bonvaloti*  *Aegithalos fuliginosus* | 100%  100%  100%  100%  100% | MN356243  KF951092  KF951090  KF951087  KF951086 |
| Strigiformes | | | | | | | | |
| *Athene noctua* | 1 | 0 | 0 | 0 | 0 | *Athene noctua* | 97.4% | MN122903 |

**Note**: number of effective sample (*N*)

**Appendix S1-Detailed methods**

**1 Dietary analysis**

**DNA Extraction**

Faecel DNA was extracted for the 1st time to identify the species of origin using QIAamp Fast DNA Stool Mini Kit(Cat. No. 51604，Qiagen). In the pretreatment prior to the 1st extraction, about 100μg of scat powder was scraped from the sample surface to reduce PCR inhibitors. A homogenization treatment was conducted prior to the 2nd extraction, and DNA was extracted for the 2nd time to identify the prey species using QIAamp PowerScat Pro DNA Kit(Cat. No. 51804，Qiagen). To avoid contamination, all extraction processes were performed over a workbench which was sterilized with 75% alcohol and wiped with nuclease removal reagent (R0123, Beyotime). Negative control was set for each batch to check the standardization of operation. DNA extract was examined by electrophoresis of 1% agarose gel, and DNA concentration, A260/A280 and A260/A230 were determined by ultraviolet spectrophotometer (NP80, Implen) before DNA extract was stored at -30℃.

**Predator identification**

Primer pairs of ATP6-F/R specific for carnivore were used (Chaves *et al.* 2011) (Appendix S1: Table S2) to identify the species of origin, which amplifies a 126bp gene fragment from mitochondrial genome. Each PCR system had 20μL, containing 2 μL DNA template; 10μL 2×*Premix Taq* (*Ex Taq* Version 2.0; Takara Biopharmaceutical Co., LTD.); 0.4μM ATP6-F/R primers; 6.4μL ddH2O (RT121-02; Tiangen Biotech). PCR reaction was pre-denaturation at 94℃ for 10 min, denaturation at 94℃ for 30s, annealing at 50℃ for 45s, extension at 72℃ for 45s, 35 cycles, and a final extension at 72℃ for 10 min. DNA extraction and PCR were performed in isolated areas.

Primer pairs of 16S-F/R (Xiong *et al.* 2016) (Appendix S1: Table S2) for vertebrate were used to further distinguish domestic dog from wolf. Each reaction system had 20μL, containing 2 μL DNA template; 10μL 2×Premix Taq (Ex Taq Version 2.0; Takara Biopharmaceutical Co., LTD.) ; 0.2μM 16S-F/R primers; 3.2μL ddH2O (RT121-02; Tiangen Biotech) and 0.4mg/mL bovine serum albumin (A8010, Solarbio). PCR reaction was pre-denaturation at 95℃ for 5 min, denaturation at 95℃ for 30s, annealing at 55℃ for 30s, extension at 72℃ for 45s, 35 cycles, and a final extension at 72℃ for 10 min.

PCR products were sequenced by SinoGenoMax Limited Company, Beijing, using a Biosystems ABI 3730XL. The corresponding sequences were trimmed and assembled in Geneious Prime 2022.0.1 (Biomatters Ltd.), and comparable sequences with a length of about 126bp were obtained. Species matching was performed using BLAST (Altschul 2012) of NCBI. When (1) the Query Coverage of the best hit reached 100%, (2) the Identical Sites reached 98% or more and (3) the corresponding species were distributed in the study area, the scat sample was considered to be from this corresponding species. If one scat sample corresponded to multiple species, the inconsistent species were excluded according to the species inventory of QNNR and “A Guide to the Mammals of China” (Smith, Yan Xie & Gemma 2010).

Appendix S1: Table S2 Primer used in this study

| Primer name | Primer purpose | Product length | Annealing temperature | (5’–3’) | Literature |
| --- | --- | --- | --- | --- | --- |
| ATP6 | Predator  identification | ~126bp | 50℃ | F: AACGAAGGTCTATTCGCCTCT  R: CCAGTATTTGTTTTGATGTTAGTTG | (Chaves *et al.* 2011) |
| 16S |  | ~350 bp | 55℃ | F: GAGAAGACCCTATGGAGC  R: ATAGAAACCGACCTGGAT | (Xiong *et al.* 2016) |
| 12SV5 | Prey  identification | ~150 bp | 60℃ | F: TAGAACAGGCTCCTCTAG  R: TTAGATACCCCACTATGC | (Tiayyba *et al.* 2011) |

**Prey identification**

The universal primers 12SV5 were used (Tiayyba *et al.* 2011) (Appendix S1: Table S2) for the prey identification, amplifying a 110bp gene segment. Primer barcodes of 6 nucleotides were added to the 5’ end of each primer. Species-specific blocking oligos (Hege & Simon 2008; Vestheim, Deagle & Jarman 2011; Shao *et al.* 2019) (Appendix S1: Table S3) were used to inhibit the amplification of predator DNA, and thus allowing the amplification of prey DNA. Three biological replicates were set for each sample. Each PCR system consisted of 25μL, including 2μL DNA template; 12.5μL 2×*Premix Taq* (*Ex Taq* Version 2.0) ; 0.1μM 12SV5-F/R primers; 1μM blocking oligo, 0.2mg/mL Bovine Serum Albumin and ddH_2_O (RT121-02; Tiangen Biotech). PCR reaction was pre-denaturation at 95℃ for 10 min, denaturation at 95℃ for 30s, annealing at 60℃ for 30s, 45 cycles, and no final extension. All PCRs were performed with a negative control (using deionized water instead of DNA templates) to check possible contamination. PCR and DNA extraction were performed in isolated areas. All experimental consumables were sterilized in a high-pressure steam cooker (120℃, 20min) and then dried in an oven (70℃, 3h). Tips with filters were used to avoid potential cross-contamination.

Appendix S1: Table S3 Blocking oligo used in this study

| Blocking oligo | Target  species | (5’–3’) | Literature |
| --- | --- | --- | --- |
| UNCiB | Snow leopard | CTATGCTTAGCCCTAAACCTAGATAGTTAGCTCAAACAAAACTAT-C3 | (Shehzad *et al.* 2012) |
| PrioC | Wolf | CTATGCTTAGCCCTAAACATAGATAATTTTACAACAAAATAATTCG-C3 | (Shao *et al.* 2019) |
| PrioC | Dog | CTATGCTTAGCCCTAAACATAGATAATTTTACAACAAAATAATTCG-C3 | (Shao *et al.* 2019) |
| PrioV | Red fox | CTATGCTTAGCCCTAAACATAAATAGTTCTATAACAAAACAATTCG-C3 | (Shao *et al.* 2019) |
| PrioL | Lynx | CTATGCTTAGCCCTAAACCTAGATAGTTAACCTAAACAAAATTATC-C3 | This study |

**High-throughput sequencing**

PCR products were first purified using *EasyPure*® PCR Purification Kit (EP101-01, TransGen), and products of same predator species were pooled together. Paired-end sequencing was performed using NovaSeq6000 (Illumina Inc., San Diego, CA, USA) by Novogene Biotechnology Co., LTD, Beijing. A total of 150 nucleotides were sequenced for each read.

**Bioinformatics process**

Raw sequence was filtered by Fastp, for any read: when N (unknown nucleic acid residue) exceeds 10% of the base number, remove the pair reads; when the number of low-quality (Quality score<=5) base exceeds 50% of the base number, remove the paired reads; When any read contains adapters, remove the paired reads. Filtered sequence was processed by QIIME 2 (2022.02) (Bolyen *et al.* 2019) package. Barcode identification (assign): QIIME ‘cutadapt’ was used to identify and compare primer barcode and primer (first 6 nucleotides), and reads were assigned to the corresponding samples allowing maximum mismatch of 1 nucleotide. Reads that failed to be assigned were discarded. Assembly, Denoise and Chimeric Removal were preformed by using QIIME ‘dada2’. Primer barcode and reads end (40-45bp) of low-quality (Quality score<=20) were removed.

Prey species identification was carried out by BLAST, according to the species inventory of the QNNR, “A Guide to the Mammals of China” (Smith, Yan Xie & Gemma 2010), “A Field Guide to the Birds of China” (Mackinnon & Phillipps 2000) and IUCN Red List. Reads of carnivores, human, or hits from non-homologous gene were excluded. One MOTU will be excluded from subsequent analysis when its reads are less than 1% of the total reads of the sample, since it is considered to be from cross-contamination (Shao *et al.* 2019). Analogically, one sample will be excluded when its reads are less than 30 since it is considered to be inaccurate to reveal dietary information.

**2 Rhythm analysis**

**Camera-trapping**

Camera stations were sited considering anthropogenic factors, local knowledge, topography (sites such as mountain ridges, the base of cliffs).Animal trails and/or carnivores signs (feces, scrapes, pugmarks, urine, and scent markings) were prefered to increase detection rate, while the unfavorable habitats for wild carnivores were avoided, such as main roads, dense townships and expansive flat river beds). A minimum Euclidean distance of 1–1.5 km was kept between each station to assure independence and reduce spatial auto-correlation, except for three stations which had distances less than 600 m due to inaccessibility. All cameras were programmed to operate for 24 h d^–1^

**Data processing**

First, we identified species of each record, and pooled together all records from the same camera station. Records taken at least 30min apart were identified as independent events to avoid spatial pseudo-replication. We used GMT+6 and coordinates of Mt.everest (27°59′17″ N; 86°55′31″E) as the timezone and the approximate location of the study area. Prior to further analysis, we adjusted clocktime of each independent event to suntime (sunrise or sunset) based on its specific date and the approximate location using ‘suntime’ function in package ‘overlap’ (Nouvellet *et al.* 2012) to take into account the successive changes of suntime throughout the year. We adopted the method presented by by Ridout & Linkie (2009) to first fit kernel density curve and then determine the overlap (the area lying under both of the density curves) between wild predators (snow leopard, wolf, lynx, red fox) and wild prey (bharal, rabbit), as well as anthropogenic factors. To obtain the 95% confidence interval of overlap, a smoothed bootstrap of 1000 iterations was operated and the basic0 output was chosen. All rhythm analyses were performed in Rstudio 2022.02.1 using the ‘overlap’ R-package (Meredith & Ridout 2021). Records that could not be identified unambiguously such as those from small mammals (i.e. pika and rat), events in low numbers (i.e. marmot, horse, mule etc.) were not included in temporal analysis.


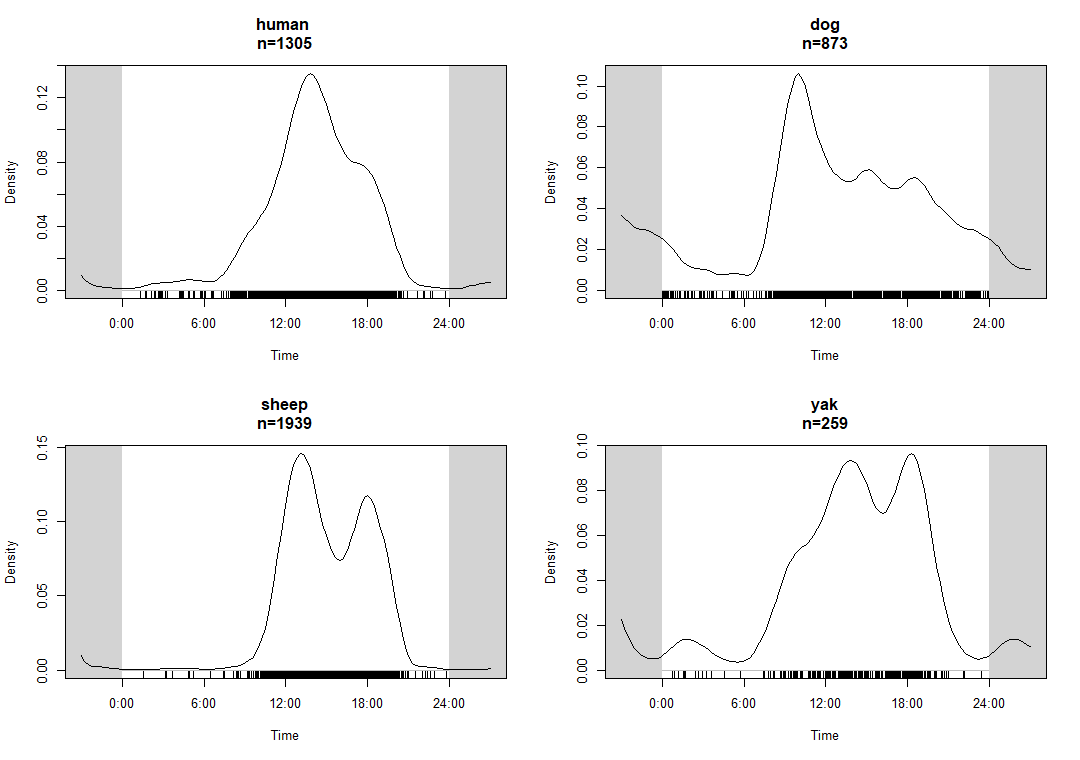


Appendix S1: Fig. S1 Daily activity patterns of anthropogenic factors (human, dog, sheep, yak) indicated by kernel density curves.

Literature cited

Altschul, S.F. (2012) Basic local alignment search tool (BLAST). *Journal of Molecular Biology,* **215,** 403-410.

Bolyen, E., Rideout, J.R., Dillon, M.R., Bokulich, N.A., Abnet, C.C., Al-Ghalith, G.A., Alexander, H., Alm, E.J., Arumugam, M., Asnicar, F., Bai, Y., Bisanz, J.E., Bittinger, K., Brejnrod, A., Brislawn, C.J., Brown, C.T., Callahan, B.J., Caraballo-Rodríguez, A.M., Chase, J., Cope, E.K., Da Silva, R., Diener, C., Dorrestein, P.C., Douglas, G.M., Durall, D.M., Duvallet, C., Edwardson, C.F., Ernst, M., Estaki, M., Fouquier, J., Gauglitz, J.M., Gibbons, S.M., Gibson, D.L., Gonzalez, A., Gorlick, K., Guo, J., Hillmann, B., Holmes, S., Holste, H., Huttenhower, C., Huttley, G.A., Janssen, S., Jarmusch, A.K., Jiang, L., Kaehler, B.D., Kang, K.B., Keefe, C.R., Keim, P., Kelley, S.T., Knights, D., Koester, I., Kosciolek, T., Kreps, J., Langille, M.G.I., Lee, J., Ley, R., Liu, Y.-X., Loftfield, E., Lozupone, C., Maher, M., Marotz, C., Martin, B.D., McDonald, D., McIver, L.J., Melnik, A.V., Metcalf, J.L., Morgan, S.C., Morton, J.T., Naimey, A.T., Navas-Molina, J.A., Nothias, L.F., Orchanian, S.B., Pearson, T., Peoples, S.L., Petras, D., Preuss, M.L., Pruesse, E., Rasmussen, L.B., Rivers, A., Robeson, M.S., Rosenthal, P., Segata, N., Shaffer, M., Shiffer, A., Sinha, R., Song, S.J., Spear, J.R., Swafford, A.D., Thompson, L.R., Torres, P.J., Trinh, P., Tripathi, A., Turnbaugh, P.J., Ul-Hasan, S., van der Hooft, J.J.J., Vargas, F., Vázquez-Baeza, Y., Vogtmann, E., von Hippel, M., Walters, W., Wan, Y., Wang, M., Warren, J., Weber, K.C., Williamson, C.H.D., Willis, A.D., Xu, Z.Z., Zaneveld, J.R., Zhang, Y., Zhu, Q., Knight, R. & Caporaso, J.G. (2019) Reproducible, interactive, scalable and extensible microbiome data science using QIIME 2. *Nature Biotechnology,* **37,** 852-857.

Chaves, P.B., Graeff, V.G., Lion, M.B., Oliveira, L.R. & Eizirik, E. (2011) DNA barcoding meets molecular scatology: short mtDNA sequences for standardized species assignment of carnivore noninvasive samples. *Molecular Ecology Resources*.

Hege, V. & Simon, N.J. (2008) Blocking primers to enhance PCR amplification of rare sequences in mixed samples – a case study on prey DNA in Antarctic krill stomachs. *Frontiers in zoology,* **5,** 12.

Mackinnon, J. & Phillipps, K. (2000) A Field Guide to the Birds of China. *Colonial Waterbirds,* **18,** 841-843.

Meredith, M. & Ridout, M. (2021) Overview of the overlap package.

Nouvellet, P., Rasmussen, G., Macdonald, D.W. & Courchamp, F. (2012) Noisy clocks and silent sunrises: measurement methods of daily activity pattern. *Journal of Zoology,* **286**.

Shao, X., Song, D., Huang, Q., Li, S. & Yao, M. (2019) Fast surveys and molecular diet analysis of carnivores based on fecal DNA and metabarcoding. *Biodiversity Science,* **27,** 543-556.

Shehzad, W., Riaz, T., Nawaz, M.A., Miquel, C., Poillot, C., Shah, S.A., Pompanon, F., Coissac, E. & Taberlet, P. (2012) Carnivore diet analysis based on next-generation sequencing: application to the leopard cat (Prionailurus bengalensis) in Pakistan. *Molecular ecology*.

Smith, E.b.A.T., Yan Xie, R.S.H., Darrin Lunde, John MacKinnon, Don E. Wilson & Gemma, W.C.W.I.b.F. (2010) A Guide to the Mammals of China. *Princeton University Press*.

Tiayyba, R., Wasim, S., Alain, V., Franois, P., Pierre, T. & Eric, C. (2011) ecoPrimers: inference of new DNA barcode markers from whole genome sequence analysis. *Nucleic Acids Research***,** e145.

Vestheim, H., Deagle, B.E. & Jarman, S.N. (2011) Application of blocking oligonucleotides to improve signal-to-noise ratio in a PCR. *Methods in molecular biology (Clifton, N.J.),* **687,** 265-274.

Xiong, M., Shao, X., Long, Y., Bu, H., Zhang, D., Wang, D., Li, S., Wang, R. & Yao, M. (2016) Molecular analysis of vertebrates and plants in scats of leopard cats (Prionailurus bengalensis) in southwest China. *Journal of Mammalogy***,** gyw061.
